# Acceptability of prepayment, social solidarity and cross-subsidies in national health insurance: A mixed methods study in Western Kenya

**DOI:** 10.3389/fpubh.2022.957528

**Published:** 2022-10-14

**Authors:** Beryl Maritim, Adam D. Koon, Allan Kimaina, Jane Goudge

**Affiliations:** ^1^Consortium for Advanced Research Training in Africa (CARTA), Nairobi, Kenya; ^2^Centre for Health Policy, School of Public Health, University of the Witwatersrand, Johannesburg, South Africa; ^3^Academic Model Providing Access to Healthcare (AMPATH), Eldoret, Kenya; ^4^Department of International Health, Johns Hopkins Bloomberg School of Public Health, Baltimore, MD, United States

**Keywords:** social solidarity, health insurance, willingness to prepay, Kenya, informal workers, mixed methods

## Abstract

**Introduction:**

Many low- and middle-income countries are attempting to finance healthcare through voluntary membership of insurance schemes. This study examined willingness to prepay for health care, social solidarity as well as the acceptability of subsidies for the poor as factors that determine enrolment in western Kenya.

**Methods:**

This study employed a sequential mixed method design. We conducted a cross-sectional household survey (*n* = 1,746), in-depth household interviews (*n* = 36), 6 FGDs with community stakeholders and key informant interviews (*n* = 11) with policy makers and implementers in a single county in western Kenya. Social solidarity was defined by willingness to make contributions that would benefit people who were sicker (“risk cross-subsidization”) and poorer (“income cross-subsidization”). We also explored participants' preferences related to contribution cost structure – e.g., flat, proportional, progressive, and exemptions for the poor.

**Results:**

Our study found high willingness to prepay for healthcare among those without insurance (87.1%) with competing priorities, low incomes, poor access, and quality of health services, lack of awareness of flexible payment options cited as barriers to enrolment. More than half of respondents expressed willingness to tolerate risk and income cross-subsidization suggesting strong social solidarity, which increased with socio-economic status (SES). Higher SES was also associated with preference for a proportional payment while lower SES with a progressive payment. Few participants, even the poor themselves, felt the poor should be exempt from any payment, due to stigma (being accused of laziness) and fear of losing power in the process of receiving care (having the right to demand care).

**Conclusion:**

Although there was a high willingness to prepay for healthcare, numerous barriers hindered voluntary health insurance enrolment in Kenya. Our findings highlight the importance of fostering and leveraging existing social solidarity to move away from flat rate contributions to allow for fairer risk and income cross-subsidization. Finally, governments should invest in robust strategies to effectively identify subsidy beneficiaries.

## Introduction

Many low- and middle-income countries (LMICs) struggle to provide adequate financial risk protection against high health care costs and are now experimenting with national social health insurance (SHI) schemes utilizing voluntary membership (1–6). These types of SHI schemes face challenges expanding membership to informal workers who have low and fluctuating incomes but constitute a large proportion of the population in many LMICs (7). The recommended path toward financial risk protection included prepayment and pooling systems where people pay before they are sick, by contributing to a pool from which they (and others contributing to the pool) can draw from in the event of sickness (5). Taking up voluntary membership of an insurance scheme requires a willingness to prepay for healthcare, and willingness to have your financial contribution to the pool benefit others who were sicker (“risk cross-subsidization”) and poorer (“income cross-subsidization”) than you, otherwise referred to as social solidarity (8).

There is growing focus on the role of solidarity in promoting participation in public health programs globally (8–11). Solidarity has been positively associated with participation in prepayment and growing financial risk pools in Europe, US and some sub-Saharan countries (8, 9, 11–16). It is founded on and influenced by trust in the scheme, and in the health systems' ability to provide a promised benefit package (17–19). Research into social solidarity can provide insight into the barriers to increasing participation in prepayment and pooling mechanisms in LMICs. In this paper, we explore the willingness to prepay and social solidarity among informal worker households in western Kenya as part of broader efforts to understand participation in SHI in similar LMIC settings.

Kenya's health sector is financed by a mix of methods with a significant contribution from private households in the form of out-of-pocket payments (OOP) (27% of the total health expenditure). Tax, donor funding, and health insurance account for 31, 25, 13% respectively (20). Kenya has averaged < 9% of its annual government budget allocation toward health in the 2014–2020 budgets (21), despite signing the Abuja Declaration, committing to a 15% allocation (22). High poverty rates (almost 60% of the population live below the poverty line) (23) reduce the capacity to collect taxation and to expand SHI contributions (24). Health insurance enrolment rates in Kenya are currently below 20% (25). The result is an overreliance on out of pocket payment at the point of service.

The Kenyan government has prioritized universal health coverage (UHC) in its policy documents such as the ‘BIG Four Agenda', Kenya's Vision 2030 and the national health policy (26). The national UHC strategy intends to use the national insurer, NHIF, as a means to increase prepayment and pooling, and increase access to health care (23). NHIF is a Kenyan government state corporation and the national social health insurer founded in 1966. It provides both mandatory membership to formal sector employees and voluntary membership to informal workers. Recent policy and legal reforms have aimed at strengthening NHIF's governance and increasing coverage especially among informal workers.

The Kenyan government has committed to gradually identifying and fully sponsoring the poorest households' NHIF membership (27). These households are identified through a poverty list developed by the Ministry of Labor, Social Protection and Services and validated at community level to ensure the program benefits the poorest (23). An impact evaluation of the health insurance subsidy program revealed challenges providing adequate coverage and significant gaps in the inclusion of households. The program has been criticized for neither achieving desired financial risk protection nor increasing access to healthcare for poorer households.

Over 80% of the Kenyan population work are informal workers (7), many running small businesses in the agricultural, food, crafts and transportation industries (28). Those working in informal economy, defined as a “parallel unregulated economy,” are not subject to official labor laws” (28), making the levying of mandatory payments for health insurance on salaries impossible; as a result, health insurance payment, and membership, is voluntary. NHIF offers voluntary membership through a scheme known as supa + cover (29).

While voluntary health insurance offers an opportunity for the informal sector households to obtain the much-needed financial risk protection, the NHIF has however struggled to attract and retain new voluntary members (30). In 2017, 73% of voluntary NHIF members did not renew their health insurance (20). Low enrolment has slowed the growth of NHIF and threatens its viability. As Kenya attempts to increase health insurance coverage through NHIF, it is important to understand the population's willingness to participate in prepayment arrangements and their views on social solidarity (31).

**Figure 1:**
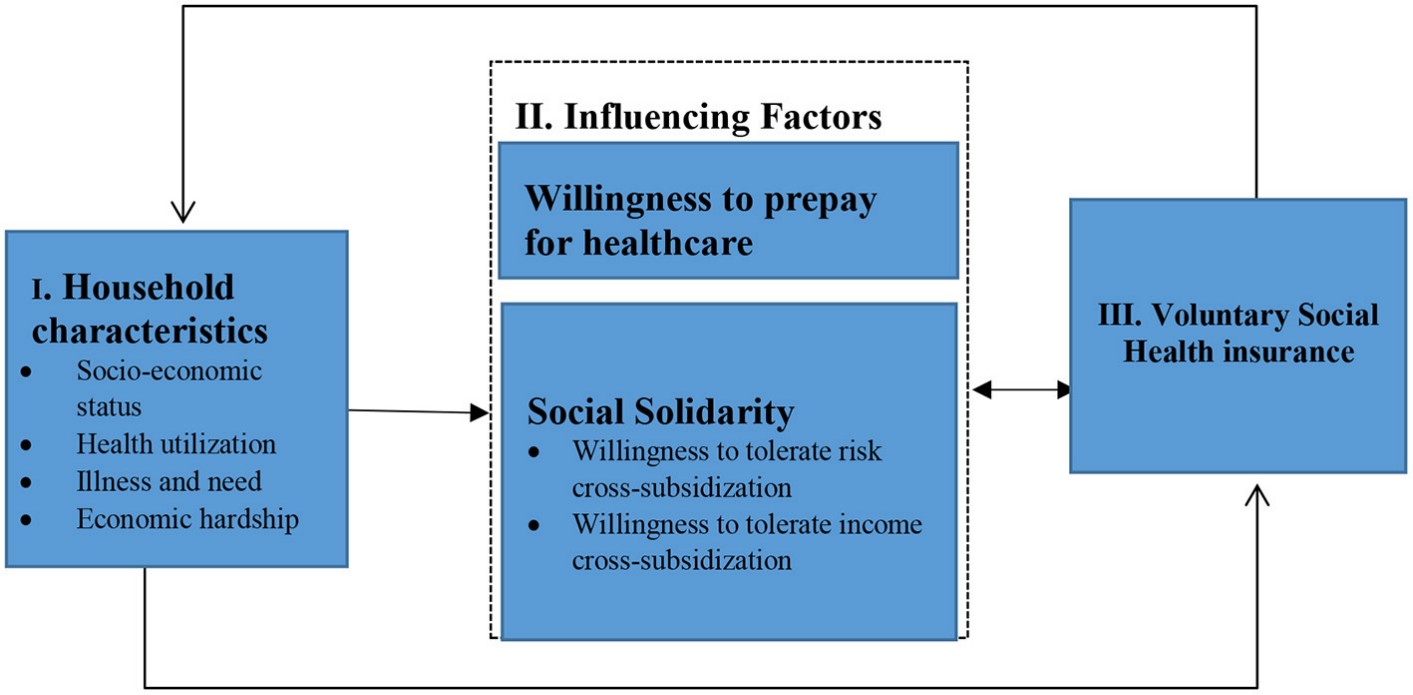
Conceptual framework on willingness to prepay, social solidarity, and health insurance.

For our study we developed a deductive conceptual framework (Figure 1) based on literature and the concepts we are focused on i.e., prepayment and tolerance of risk and income cross-subsidies. Household characteristics such as socioeconomic status, age, health and education influence views on willingness to prepay and social solidarity (13). Social solidarity and household characteristics are in turn hypothesized to influence voluntary health insurance uptake (8, 19). This paper is part of a larger PhD study exploring the role of affordability and citizen empowerment and other determinants on health insurance enrolment, the findings of which will be reported elsewhere.

## Methods

### Design

This was a mixed methods study with sequential qualitative and quantitative primary data collection (32). The study was part of a broader cross-sectional baseline household survey conducted by Academic Model Providing Access to Health care (AMPATH) between August and September 2021.

### Study site

The study was conducted in Bunyala sub-county, in Busia country, located on the western border with Uganda. Poverty levels are among the highest in the country with poor health indicators and low health insurance enrolment (33, 34).

### Study participants

We interviewed household respondents (>18 years) with adequate knowledge on households spending and healthcare utilization (Table 1) (35). Households were selected from an existing program database containing all households in Bunyala sub-County. The 6 locations in Bunyala sub-county formed the stratum for household sampling. A household list was prepared from the database and was used to randomly select households to participate in the survey under each stratum (*n* = 1,746). We asked all respondents whether they would be willing to participate in a subsequent qualitative interview.

**Table 1:** Data collection method and study participants.

**Interview method**	**Number of interviews**	**Participants**
Household questionnaire	1,746	Household respondents (>18 years)
Household in depth interviews	36	Household respondents (>18 years)
Focus group discussions	6 groups with a total of 49 participants (30 male and 19 female)	Community stakeholders/representatives
Key informant interviews with policy makers and implementers	11	Sub-County, County and National policy makers, and implementers

For the in-depth interviews, we identified households who had participated in the quantitative phase, had been able to provide health care utilization and expenditure data, and had said they were willing to participate in a second interview. From this group, for each of the 6 administrative locations in the study site, we purposively selected two households from each of the following two groups: (a) currently enrolled in NHIF, (b) previously enrolled in NHIF, and (c) never been enrolled in NHIF.

For the FGDs, we purposively recruited 6–8 community stakeholders. These were mostly community opinion leaders, local administration, CSO members, community health volunteers (CHVs), ward administrators, youth representatives, teachers and religious leaders identified based on their active advocacy on health insurance related issues. We conducted 6 FGDs, one in each of the 6 sub-location with a total of 49 participants (30 males and 19 females). Key informant interview participants were also selected purposively from policymakers and implementers at facility, sub-county, county and national levels through snowballing methods (*n* = 11).

### Data collection

#### Quantitative

Trained interviewers collected the survey data using a structured questionnaire. With the help of local administration, data collectors with experience in national survey data collection were identified locally. The interviewers were trained on the use of tablet-based questionnaires and on the baseline survey concepts, procedures and guidelines (13, 36). Interviewers were paired with CHVs whose roles were to navigate the area and introduce the interviewers to the households. Interviews were confidential and CHVs did not take part in the interviewing session. The survey collected household information on household size, education level, health insurance status, health status, marital status, income level, household spending and healthcare utilization, health status, common illness, willingness to cross-subsidize and prepay for healthcare. Questions on social solidarity that were included in the questionnaire are adapted from the surveys previously carried out in Ghana, Tanzania and South Africa where they used show cards to assess income cross-subsidization and asked question on prepayment and social solidarity (13). Data collection tools were pretested in piloted and modifications made to address problems and potential errors. Potential challenges with the local interpretation of the show card led to some adjustments to fit the study setting context. In addition, we mitigated the effects of social desirability bias by triangulating data collection techniques to validate essential variables, using trained and skilled interviewers, and pilot-testing the tools. Survey data collection was conducted between August and October 2021.

#### Qualitative

All interviews were conducted in person with KIIs with policymakers taking place in the respondent's choice of location, household interviews in respondent homesteads while FGDs were conducted at the nearest health facilities or community space. The researcher (BM) conducted interviews with the policymakers and facilitated half the FGDs. The rest of the interviews were facilitated by 3 trained research assistant. We explored respondents' views on social solidarity through semi-structured qualitative interviews using interview guides. BM, JG, and AK held debriefing sessions after a batch of interviews to identify areas of improvement in the exercise. KIIs and household IDI interviews took an average of 30–40 min while the FDGs were between 2 and 2.5 h. We conducted qualitative interviews between August and October 2021.

#### Assessing willingness to prepay, social solidarity

We measured willingness to prepay among the respondents by asking whether they would agree to pay a small amount each month so that if they got sick, health care will be free. To measure social solidarity, we assessed the respondent's tolerance of income and risk cross-subsidization, we asked whether they would be willing to contribute the same amount of money each month as everyone else, even though others who are more poor or sick than them will use the services more. This question was related to cross-subsidization that happens in financial risk pools.

**Figure 2:**
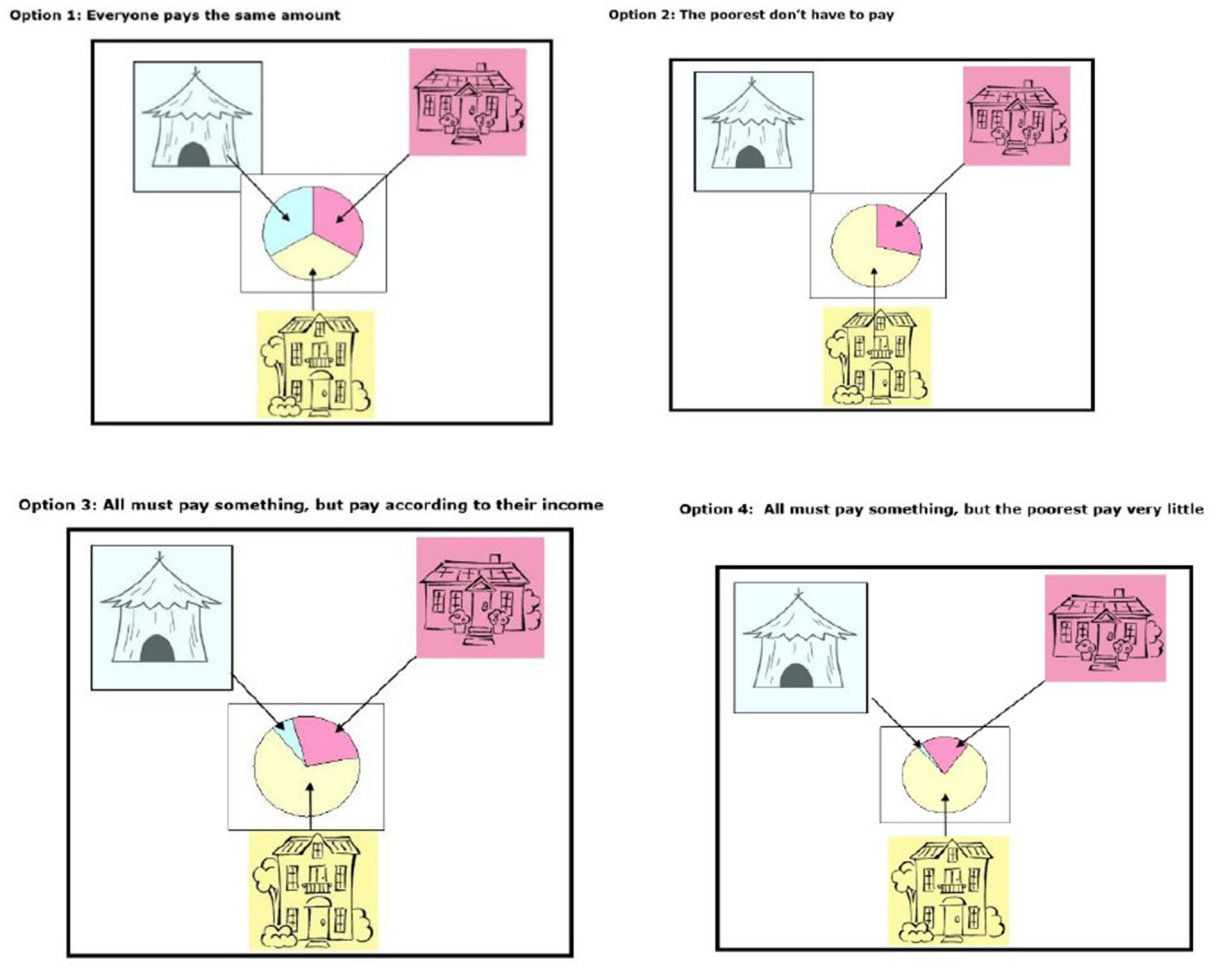
Show cards used in the study to determine preference of payment method adapted from Goudge et al. (13).

To assess the determine their preference for different relative contributions that offers desired cross-subsidization, we presented household respondents with four healthcare contribution options in the form of pictorial diagrams (show cards) with housing type as the form as measure of socio-economic status (SES) (Figure 2). The four option were (1) everyone pays the same amount (flat contribution); (2) everyone must pay according to their income (proportional contributions e.g., everybody pays 5% of their income irrespective of their income level); (3) all must pay something, but the poorest pay very little (all pay progressive contributions, where the percentage increases with income); (4) progressive contributions but the poor do not pay. Respondents were then asked to select one that resonated with their belief of how healthcare contributions should be made.

### Analysis

In the quantitative analysis, we first conducted descriptive analysis to examine willingness to prepay, and tolerate income and risk cross-subsidization by the socio-demographic factors identified in literature (13). Secondly, we used bivariate logistic regression to examine the influence of population characteristics on the three outcomes of interest -willingness to prepay, and tolerate income and risk cross-subsidization. Odds ratio (OR) and corresponding confidence intervals were used to report the strength of influence of the socio-demographic factors on the 3 outcomes. The analysis was presented at 95% significance level at α = 0.05. The analyses set the reference group for OR (odds ratio) as 1.0. Data analysis was done using R and relevant R packages.

The audio recordings from the IDIs with household heads, KIIs with policymakers and implementers, and FGDs were transcribed manually using Microsoft word and compared against their respective audio files for accuracy. Qualitative data was analyzed manually using a thematic framework analysis approach (37). BM read through the transcripts to familiarize with the data first. Second BM, JG, and AK began identifying key ideas and phrases from a few sampled transcripts and developing labels or codes. Once the team agreed on the labels or codes, BM went ahead and applied them to subsequent transcripts. A mind map was then used to categorize the codes based on the study objective and conceptual framework. The main categories were social solidarity and pooling, willingness to prepay, barriers to prepayment and views on preference of payment approaches. In an iterative process, we drew relationships between the categories to identify and refine themes. Lastly, we interpreted the results and presented them under each theme and included supportive quotes from the interviews.

### Ethical consideration

Ethical approval was obtained from University of Witwatersrand (Certificate no. M210216) and the Moi University/Moi Teaching and Referral Hospital Institutional Research Ethics Committee (IREC)-(No. 0003935), before beginning the study. Additional approval was obtained from the National Commission for Science Technology and innovation (NACOSTI-P/21/12262) and Busia County Health Department. Written informed consent were also obtained from participants willing to participate in the study.

## Results

### Household survey findings

#### Willingness to prepay

Nearly all the respondents expressed willingness to prepay for their healthcare costs (88%) (Table 2). Respondents expressing willingness to prepay were 4.09 times likely to be aged 25–40 compared to those above 65 years. Higher likelihood of expressing willingness to prepay was recorded among respondents those who had attended school (OR2.82), those of higher SES (OR 4.07 and 3.12 for Q4 and Q5), and those with health insurance (OR 3.45). Interestingly, 87.1% of the respondents without health insurance expressed willingness to prepay. Also unexpectedly, lower likelihood of expressing willingness to prepay was recorded among respondents with chronic illness (OR 0.64). Lower likelihood was also reported among informal workers (OR 0.4). All these associations were statistically significant.

**Table 2:** Willingness to prepay and tolerance of risk cross-subsidies among the respondents.

			**Willingness to prepay** ^**b**^				**Income cross-subsidies** ^**c**^				**Risk cross-subsidies** ^**d**^		
**Characteristic**		**%(*n*)**	**OR^a^**	**95% CI^a^**	***p***	**%(*n*)**	**OR^a^**	**95% CI^a^**	***p***	**% (*n*)**	**OR^a^**	**95% CI^a^**	***p***
Age	18–24	90.7 (714)	2.65	(1.43, 5.31)	**0.003**	55.4 (439)	0.91	(0.57, 1.44)	0.7	61.1 (483)	0.86	0.54, 1.37	0.5
	25–44	91.4 (551)	4.09	(2.94, 5.73)	**<0.001**	57.5 (347)	1.3	(1.01, 1.68)	**0.039**	65.0 (392)	1.51	1.17, 1.94	**0.002**
	45–64	73.5 (202)	3.89	(2.75, 5.54)	**<0.001**	49.5 (136)	1.47	(1.13, 1.91)	**0.004**	52.4 (144)	1.79	1.37, 2.34	**<0.001**
	?64	85.2 (69)	—			45.7 (37)	—			49.4 (40)	—	—	
Sex	Female	63.0 (967)	—			52.3 (581)	—			58.2 (647)	—	—	
	Male	89.6 (569)	1.1	(0.83, 1.46)	0.5	59.5 (378)	1.4	(1.16, 1.69)	**<0.001**	64.9 (412)	1.37	1.13, 1.66	**0.001**
Marital status	Divorced/separated	90.5 (134)	—			54.1 (80)	—			60.8 (90)	—	—	
	Married/ living together	90.4 (989)	0.87	(0.47, 1.50)	0.6	56.1 (614)	1.12	(0.79, 1.58)	0.5	61.9 (677)	1.06	0.75, 1.50	0.7
	Never married/ never lived together	78.3 (90)	0.45	(0.22, 0.90)	**0.026**	54.8 (63)	1.32	(0.82, 2.13)	0.2	60 (69)	1.22	0.75, 1.99	0.4
	Widowed	83.0 (323)	0.49	(0.26, 0.88)	**0.022**	51.9 (202)	0.91	(0.63, 1.33)	0.6	57.3 (223)	0.84	0.57, 1.23	0.4
Ever attended school	No	79.7 (278)	—			49.0 (171)	—			52.1 (182)	—	—	
	Yes	90.1 (1,258)	2.82	(2.12, 3.75)	**<0.001**	61.9 (788)	1.5	(1.20, 1.88)	**<0.001**	99.6 (877)	1.77	1.42, 2.22	**<0.001**
Occupation	Formal employment	95.3 (41)	—			76.7 (33)	—			81.4 (35)	—	—	
	Homemakers (Stay at home)	83.5 (232)	0.19	(0.03, 0.62)	**0.022**	46.4 (129)	0.26	(0.12, 0.52)	**<0.001**	53.6 (149)	0.25	0.10, 0.52	**<0.001**
	Students	57.1 (4)	0.06	(0.01, 0.46)	**0.008**	57.1 (4)	0.37	(0.07, 2.13)	0.2	42.9 (3)	0.16	0.03, 0.84	**0.031**
	Unemployed/Seeking work	78.5 (84)	0.17	(0.03, 0.60)	**0.018**	54.2 (58)	0.33	(0.14, 0.71)	**0.006**	59.8 (64)	0.33	0.13, 0.73	**0.01**
	Working in informal employment (e.g., farmers, artisans, juakali, business etc.)	89.8 (1,167)	0.4	(0.06, 1.30)	0.2	56.2 (731)	0.37	(0.17, 0.72)	**0.006**	61.7 (802)	0.35	0.15, 0.71	**0.007**
	Others (Specify)	72.7(8)	0.12	(0.01, 0.84)	**0.033**	36.4 (4)	0.16	(0.04, 0.63)	**0.011**	54.5 (6)	0.25	0.06, 1.06	0.056
Have health insurance	No	87.1 (1,345)	—			53.3 (824)	—			58.5 (904)	—	—	
	Yes	95.0 (191)	3.45	(1.90, 7.06)	**<0.001**	67.2%(135)	2.03	(1.51, 2.76)	**<0.001**	77.1%(155)	2.76	1.99, 3.91	**<0.001**
Wealth quintile	Q1	79.5 (276)	—			46.4 (161)	—			52.7 (183)	—	—	
	Q2	85.1 (298)	1.67	(1.16, 2.42)	**0.006**	56.9 (199)	1.74	(1.31, 2.30)	**<0.001**	60.9 (213)	1.54	1.16, 2.05	**0.003**
	Q3	90.9 (311)	2.37	(1.58, 3.62)	**<0.001**	51.5 (176)	1.25	(0.94, 1.67)	0.12	56.4 (193)	1.17	0.88, 1.57	0.3
	Q4	92.9 (326)	4.07	(2.58, 6.63)	**<0.001**	53.8 (189)	1.47	(1.11, 1.95)	**0.007**	63.2 (222)	1.66	1.25, 2.22	**<0.001**
	Q5	91.3 (325)	3.12	(2.03, 4.90)	**<0.001**	65.7 (234)	2.19	(1.64, 2.94)	**<0.001**	69.7 (248)	2.03	1.52, 2.74	**<0.001**
Member financial group chama	No	86.0 (928)	—			52.5 (566)	—			58.5 (631)	—	—	
	Yes	91.2 (608)	1.84	(1.37, 2.51)	**<0.001**	58.9 (393)	1.16	(0.97, 1.40)	0.11	64.2 (428)	1.16	0.96, 1.40	0.13
Admitted last 12 m	No	88.0 (1,435)	—			54.9 (895)	—			60.3 (983)	—	—	
	Yes	87.8 (101)	0.93	(0.56, 1.63)	0.8	55.7 (64)	1.06	(0.74, 1.54)	0.7	66.1 (76)	1.3	0.89, 1.92	0.2
Chronic ailment	No	89.9 (842)	—			53.3 (499)	—			60.0 (562)	—	—	
	Yes	85.5 (694)	0.64	(0.49, 0.83)	**0.001**	56.9 (460)	1.11	(0.93, 1.33)	0.3	61.4 (497)	1.01	0.84, 1.22	0.9
		**88.0 (1,536)**				54.9 (959)				60.7 (1,059)			

a OR, Odds Ratio; CI, Confidence Interval. The bold *P* values are statistically significant at α = 0.05.

b I would agree to pay a small amount each month so that if I get sick, health care will be free, even if I am not sick now.

c I would be willing to pay the same amount of money each month as everyone else, even though others who are more sick than I am will use the services more than me.

d I would be willing to willingness to pay the same amount of money each month as everyone else, even though others who are more poor than I am will use the services more than me.

#### Social solidarity-tolerance of income and risk cross subsidization

Just over half of the sample (54.9%) were in favor of income cross-subsidies(IC) (Table 2) while nearly two thirds of the respondents (60.7%) were willing to tolerate risk cross-subsidization (RC). A higher likelihood of tolerating both income and risk cross-subsidies was expressed by the male respondents (IC: OR 1.4 and RC: 1.37). Higher likelihood of expressing tolerance for cross-subsidization was recorded among respondents those who had attended school (IC: OR 1.5, RC: 1.77), those of higher SES (IC: OR 2.19 and RC 2.03 for Q5) and those with health insurance (IC: OR 2.03 and RC: 2.76). All these associations were statistically significant. Seemingly, tolerance of cross-subsidization increased with wealth. Lower likelihood was also reported among informal workers (OR 0.4) compared to those in formal employment and the divorced (OR 0.91, 0.84) compared to the married. Only 67.2% of those with health insurance were willing to cross-subsidize those who are poorer than themselves indicating a poor understanding of insurance.

#### Preferences for different relative contributions

Nearly half of the respondents (41.2%) were in favor of a progressive payment while only 4.9% of the respondents believed that everyone should pay the same amount (Table 3). The most popular options among the wealthy were the proportional payment (38.8%) and the progressive where the poor don't pay at all (23.1%). Surprisingly, among the poorest, progressive payments was the most popular option (54.2%) with option where the poor don't pay being much less popular (17.6%).

**Table 3:** Payment Preference and wealth quintiles of the respondents.

**Wealth quintiles**	**Progressive but the poor don't pay** ***n* = 399**	**Progressive** ***n* = 720**	**Proportional** ***n* = 541**	**Same flat amount** ***n* = 86**	***p* ^a^**
	**%(n)**	**%(n)**	**%(n)**	**%(n)**	
Q1 (Poorest)	17.6 (61)	54.2 (188)	19.9 (69)	8.4(29)	<0.001
Q2	26.0 (91)	41.4 (145)	27.7 (97)	4.9 (17)	
Q3	22.5 (77)	43.9 (150)	30.7 (105)	2.9 (10)	
Q4	22.2 (78)	36.5 (128)	37.6 (132)	3.7 (13)	
Q5 (wealthiest)	25.8 (92)	30.6 (109)	38.8 (138)	8 (17)	
Total (%)	22.9 (399)	41.2(720)	31.0 (541)	4.9 (86)	

### Qualitative findings

#### Willingness to prepay and understanding of pooling

Respondents expressed a preference to make small regular contribution in advance to access free healthcare when they needed it. One of the perceived benefits of prepayment observed by participants is that it helped people access care without delays: “*Sometimes one is in a critical condition and the treatment is required urgent. At that point, there is no time to sit down as a community and fundraise and fundraise. That can take time.” (FGD F* R4). Most of the respondents cited the rising cost of treatment as well as higher incidence and unpredictability of illness as the main reason to prepay: “*It is easier to put money in a pot every month, so you can get free services when you need it, than you pay when you are sick. Diseases come when there is no money. (FGD C R2)*.

Despite the recognized importance of prepayment, many households were not enrolled into any health insurance scheme and paid for their healthcare at the point of service. Lack of awareness of available insurance mechanisms was cited as the reason for not enrolling. Community representatives and policy makers also noted that majority of the population comprised of low-income earners with many competing priorities and healthcare did not rank top of the priorities especially for the youth: “*We are all different and all have different priorities on how to spend our income. You can't pay for insurance and yet you do not have food.” (FGD F* R4) Respondents expressed the desire to make smaller and flexible contributions toward premiums.

### Trust

Trust in the health system was repeatedly mentioned by in the interviews. Reliability and quality of services was a key consideration for making decisions to enroll for insurance. Respondents expressed mistrust in the health care delivery system citing negative attitude and discrimination against the poor. There were complaints of unfair treatment and unreliable services often discouraging prepayment or causing many to default from making regular contributions: “*There are people with diabetes and blood pressure who are always contributing for health insurance but when they get unwell and come here for service, there are no drugs. Will they continue to contribute? They will decide they would rather give cash and get services.”* (*FGD F* R4) Geographical distribution of health facilities was also a concern. Participants felt that the pro-urban distribution of health facilities and the quality of services favored the urban dwellers and discouraged enrolment of rural populations.

#### Social solidarity and risk and income related contributions

The sense of community was central to cultural identity and individuals felt bound together by this understanding*: “Within the African set up especially, we really tend to identify ourselves with our neighbors and our relatives. In as much as we will want to think about ourselves, most of the things we do in society usually involves others.” (KII_033)* Harambees (fund raisings) were the commonest form of solidarity often relied on for both financial and social security in times of need: “*Even in common culture, fundraisings are part of our culture to raise money when people are unwell or sick. I think there is a common thread running through that people do support each other.” (KII_09)* However, respondents observed weakening ties evidenced by declining participation in fundraisings: “*Actually when I look at the society now, it is like the issue of harambee is becoming very unpopular. There were years you used to call a harambee, and you would see a crowd. Nowadays you don't see that. Maybe people are fatigued, and they have better ways of dealing with their issues.” (KII_11)*

For many of the respondents, the health of the family formed the priority, with some participants expressing unwillingness to contribute to healthcare costs of people beyond their immediate family. “*If we said that we will pay for insurance that will cover the neighbors, we will have lied to ourselves” (FGD F* R6*)*. Feelings of mistrust were expressed between members of the community with regards to their willingness to support others. Even when people declared good intent, they did not always honor them: “*Many times we hear someone saying at a burial, “These children belong to my brother. I will do this and this for them” but they never do it. When they leave, they are gone completely.”* (FGD F R2).

Tolerance for risk and income cross-subsidization varied across the respondents. Some respondents expressed regard for the plight of others in the community especially those who were sicker revealing a higher to tolerance of risk cross-subsidization: “*Maybe you are the kind who is blessed and you never get sick, yet you have a neighbor who is always sick; if you helped in this case, isn't that a good thing?”* (FGD F R1). Income cross-subsidization received relatively less support. The idea that contributions could benefit those who were poorer was viewed as burdensome by some respondents: “*If we say we carry each other on the back, we will not be helping each other. It is good for everyone to be self-reliant (FGD C R6)*. Respondents expressed particularly higher distrust toward the rich who were otherwise viewed as having acquired their wealth by exploiting the poor. These were followed by concerns that they would not agree to cross-subsidize the poorer because of their “cruel” nature.

Policy makers expressed concern that there was limited awareness and understanding of how insurance pools and often, when people contribute to insurance, they are not aware of the cross-subsidization element: “*When they pay premiums, they won't really be thinking that it would pay for someone else, and they might actually be displeased to know that it happens in practice.” (KII_09)* A representative of NHIF further elaborates on an expectation that the pool should cover all medical cost revealing a lack of awareness on their entitlements and services covered in the benefit package. “*When most people contribute, they think we bank for them. When one has an accident and doesn't get approval for the surgery, they will ask, “Why? I have been being contributing all this time and I have not used this money. Who has been using it?” (KII_02)* There was need to sensitize the community on existing prepayment and pooling mechanisms as well as the benefit package to increase participation: “*Once people understand the importance of having a pool of resources to benefit the entire community, then it becomes cheaper for everybody.” (KII_04)*.

#### Preferences for different relative contributions

The flat payment method option was the least preferred approach because it allowed the least level of cross-subsidization and denied access to the poor. This option used by NHIF was the main reason many people didn't renew their membership: “*Today I may get Ksh 1,000 or Ksh 500 for this whole month; will I go to pay NHIF and my kids to sleep hungry? Of course not! So, I skip paying for that month. Once two months pass, I will become demotivated.” (FGD D R3*). Respondents felt that it was not fair to impose equal contributions across people of different socio-economic status: “*You see not everyone is equal because what you earn is not same as what I earn, so we cannot pay the same.” (IDI_A_Never_01)*. However, others objected to contributions based on ability to pay because the same principle was not applied outside the health sector: “*If you go to shops, they haven't differentiated the price of items for the rich and the poor. If our wish is to be treated the same, I am for the opinion that the rates to be the same.” (FGD B_R4)* Some participants believed that contributions be based on need not income but others believed that all should pay the same amount since sickness affected both the rich and the poor.

#### Should the poor pay?

The idea of exempting the poor elicited mixed views. Most of the community respondents feared that exempting the poor from making contributions would create dependency or condone laziness. Ensuring everyone contributed enforced a culture of hard work and eliminated the culture of receiving handouts: “*Free things bring laziness. People don't see a reason to work. They can be subsidized but it should not be completely free. (FGD D R4).” (FGD D R6)* Some said that contributing promoted a sense of ownership, arguing that it would give everyone, and especially the poor, the right to demand for quality services. There was a recognition that healthcare was expensive, and respondents held the view that it was important to mobilize contributions from everyone as one policy maker stated*: “You know financing health is very expensive. If we say we will pay for the poor, we may not sustain it.” (KII_01)* Respondents also argued that the poor placed a higher burden on the health system because they were a majority and often sicker, and therefore had to contribute.

In contrast, respondents who identified themselves as poor especially expressed the desire to be exempted and felt that the rich needed to contribute more in order to cross-subsidize the poor: “*I'd like them to pay for me, because if you tell me to pay from my income, I could not afford it.” (IDI_F_Never_01)*. A few respondents felt that the poor could not afford to pay for healthcare and should be exempted from contributing and therefore supported subsidizing the poor. However, government processes to determine subsidy recipients were often not transparent. Respondents expressed mistrust in the government institutions' ability to objectively determine who to exempt*: “We see that we have government cash transfers. It has left out those people who have no means. And it has enrolled other people. Those with connections benefit while those people who have no means are left out.” (FGD D R1)*

## Discussion

Our study revealed that there was a high willingness to prepay for health care among those without insurance but competing priorities, low incomes, poor access and quality of health services, lack of awareness of flexible payment options were cited as barriers to enrolment. While more than just over half of the respondents expressed willingness to tolerate risk and income cross-subsidization, suggesting a strong sense of social solidarity, that increases with socio-economic status. However, the wealthy were more in favor of a proportional payment, and the poor, a progressive payment, although there seemed to be limited understanding of the importance of pooling funds. Despite many reporting they could not afford to pay the membership fee, only less than one fifth of the poor (17.1%) felt the poor should be exempt. This may be due to stigma (being accused of laziness) and fear of losing power in the process of receiving care (having the right to demand care). Our findings suggest the concept of social solidarity was consistent with the historical African culture of standing with one another but over time the level of social solidarity was observed to be diminishing.

Our findings on social solidarity reveal a higher willingness to tolerate risk cross-subsidization compared to income cross-subsidization and was observed across each wealth quintile. This demonstrates a higher consideration for the sick than for the poor. The observed difference may also be indicative of the population's low tolerance of income cross-subsidies as poverty is perhaps thought to be a result of an individual's efforts. Indeed, some participants felt that contributing to healthcare cost of others was burdensome, unrealistic and the reluctance to contribute was justified by the need to promote self-reliance among the poor. These findings could explain rising opposition to attempts in Kenya to revise premiums for formal sector workers in order to cross-subsidize the poor. Such proposals were met with significant opposition by labor unions who preferred not to bear the responsibility of the poor (38). Similar views and opposition to policy reforms favoring the poor were recorded in a study in other LMIC settings and were found to limit social solidarity (39, 40). The objective of pooling prepaid contributions is to redistribute financial risk associated with healthcare among members of the society and therefore solidarity and support for risk income and risk cross-subsidization provide a conducive environment for pooling in the context of voluntary enrolment into health insurance (1, 13, 17, 30, 41).

Willingness to tolerate cross-subsidization increased with an increase in wealth and education implying a better understanding of pooling principles among the educated and those in higher SES groups. For example, likelihood of expressing willingness to tolerate income and risk cross-subsidization was higher among the highest quintile(richest) than the lowest quintile (poorest). Sixty-nine percent of respondents in the highest quintile(richest) expressed support for risk cross-subsidization compared to 52.7% of the lowest quintile(poorest). These findings are consistent to the results of the SHIELD Study in south Africa where greater support for risk cross-subsidization was reported among the higher income groups (65%) compared to the lower income groups (48%) (13). It is plausible that willingness to cross-subsidize was corresponded with the ability of the individuals to contribute and therefore lesser ability to contribute led to less willingness to cross-subsidize.

Kenyans have long been pooling their money to protect themselves against the financial risks arising from high medical expenses. The culture of fundraising, commonly referred to as “harambees” is a common form of solidarity and dates back to African cultural construct where solidarity among community and family formed part of individual identity. Similar findings are documented in other studies in South Africa where the African Ubuntu philosophy promoted communal relationships and mutual consideration for others (23). Despite operating on similar principles of contribution and pooling, the culture of raising funds through harambees seems more acceptable than health insurance. This could be explained by the fact that harambees, tend to happen among smaller social networks where the beneficiaries are identifiable and do not require long term financial commitment. Achieving the same level of solidarity among a much wider, diverse population nationwide, for social health insurance is harder. However, despite the popularity and reliance on harambees as an informal financial security, our findings reveal concerns that the tradition of fundraisings was declining due to modernization and socioeconomic changes. With the weakening of social ties and hard economic times globally, this form of solidarity can no longer be relied on to provide pooling and financial protection as it historically did, warranting the expansion of enrolment into health insurance (19). Higher levels of cross-subsidization can be achieved with nationwide pooling systems like health insurance compared the cross-subsidization of a small group of poor rural people sharing their limited resources. Nationwide pooling draws the rural poor into the same pool as the wealthier urban population.

Our findings show that poverty is a significant barrier to increasing prepayment through health insurance. Despite a high willingness to prepay for healthcare, and the willingness to cross subsidize, the ability to pay for health insurance was a significant barrier to participation in schemes. This is evidenced by results from the recent Kenya Household Health Expenditure and Utilization Survey (KHHEUS) 2018 where 42% health insurance enrolment was reported among higher wealth quantiles while only 2.9% of the lowest wealth quantiles have insurance (30). Efforts to increase participation in health insurance schemes need to ensure the poorest are supported to access insurance through subsidy programs. Our study participants expressed concerns and fears of discriminatory treatment against the poor and the skewed distribution of resources, information and health facilities in favor of the wealthier populations. Health facilities were believed to discriminate against people of lower SES, offering them substandard care. Participants demanded equitable access and treatment across SES as a condition to enroll in prepayment schemes. Indeed, previous studies reveal that people of higher SES experience better healthcare than middle and lower SES (11, 42).

Our study also revealed the difficulty of exempting the poor from making contributions because of the difficulties of determining income levels among informal workers. Recent global and national UHC policy reforms are anchored on the need to prioritize the needs of the poorest and vulnerable in order to achieve truly “universal” coverage (43). This raises a pertinent question on the best way to determine SES to exempt the poor. Programs meant to benefit the poor have often failed to have the desired impact because of reported errors in inclusion inaccuracies (42, 44–46). The difficulties in measuring poverty among informal workers is a major interest in many program areas (47). The poor constitute a large proportion of the population in LMICs. According to the recent national household survey on, it is estimated that about 60% of Kenyans live below the poverty line making the issue of poverty especially important (23). Respondents in our study expressed concerns that the poor placed a high burden on the country's health sector. There were also concerns about exempting the poor from making payment promoted laziness. This implies a reluctance to cross-subsidize the poor.

Various dimensions of trust were found to contribute to willingness to prepay and level of social solidarity as revealed by our study findings. These dimensions include trust in the public health system, trust among scheme members, and trust in the government institutions to objectively target vulnerable households to benefit from insurance subsidy program. Respondents based decisions to participate in prepayment schemes on perceived trust in the health system. They expressed mistrust in the formal public health system citing unreliability of services, poor services offered in facilities, negative staff attitudes, discrimination of the poor in healthcare delivery and pro-urban distribution of facilities. These views are validated by findings of previous studies that found that trust in the health system influenced participation in insurance schemes (14, 18, 19). Perceived mistrust of government systems used in means testing to exempt the poor from contributing had failed in other programs. This is confirmed by other studies that found that 65% of the national health insurance subsidy recipients meant for the poor belonged to wealthier quintiles (42). This is indicative of a targeting challenge that had led to mistrust of government systems used to establish vulnerability among community members-a common problem in many LMICs. Similar targeting challenges are reported in Ghana's National Health Insurance Scheme (NHIS) (13, 48). Efforts to increase coverage must therefore be accompanied by strategies to address trust and reliability of health services and systems.

## Recommendations for policy

Our findings have a number of policy implications in Kenya and similar LMIC settings. First, social health insurance schemes have the opportunity to foster and leverage existing solidarity to increase participation in health insurance programs. Solidarity in insurance can be strengthened by ensuring better understanding of the concept of risk pooling. For instance, messaging about NHIF should deliberately frame the scheme as a central pot that all members benefit from and the premium contributions to the risk pool as individual contributions to the good of the community.

Second, trust in the health system and NHIF should be strengthened through improving communication and relationships between the community, healthcare providers, and NHIF. Citizen involvement and engagement is a key element in building trust. Policies that facilitate community engagement should be adopted and forums for citizen engagement by NHIF and the ministries of health should be established. To curb the low awareness on prepayment that persists, NHIF should focus efforts in creating awareness on NHIF processes, and health services covered by NHIF. There is also need for continuous monitoring of service delivery by NHIF accredited providers to improve quality of services. Lastly, there is need to build trust and transparency in the identification of beneficiaries for national social assistance programs including the national health insurance subsidy program. An important step to this is community verification of vulnerability as part of the identification process.

## Limitation of the study

A key limitation of previous inquiries into the subject of social solidarity in health insurance is that they have been mostly quantitative and therefore did not describe the respondents reasoning underlying their responses on social solidarity. The mixed method study approach assumed in our paper attempts to bridge this limitation. This study took part in a small geographical area made up of a relatively ethnically homogenous population that is predominantly poor and should be interpreted with this consideration in mind. The results of our study can be generalized to other rural areas with low SES and informal workers. Further researcher would be required among urban population and among individuals of higher SES. We also acknowledge that other health economics methods have been used to explore willingness to pay. The goal of our paper was to get a broad understating on the risk and income distribution preference in the society. We did not take the willingness to pay approach on willingness to prepay and tolerance of cross-subsidization because we wanted to focus respondents' acceptance of the principle of prepayment and pooling. This is an important consideration for insurance coverage and presents preliminary work that further research can build off.

We therefore caution that these proportions may not translate to decisions to prepay for healthcare as they may be influenced by hypothetical bias. Hypothetical bias occurs when participants overstate hypothetical preference different from actual preference. Despite our best efforts to minimize their effect, social desirability and hypothetical bias may have led respondents to overstate willingness to prepay or cross-subsidize. Nonetheless we believe, our study is a novel exploration into the social solidarity, prepayment of healthcare and health insurance among informal workers. We presented findings that help us understand the role of health insurance related factors in determining health insurance enrolment. Further research is needed to the role of social solidarity on health insurance enrolment.

## Conclusion

This paper assessed the level of social solidarity and willingness to prepay as a key consideration in gaining broader understanding of participation in social health insurance programs. We explored how individual socio-economic and health status influenced their views on willingness to prepay and social solidarity. Although there is a high willingness to prepay for healthcare, low incomes, inadequate awareness of prepayment mechanisms and poor service delivery at health facilities hinder voluntary enrolment into existing SHI schemes. Efforts to increase awareness would need to be coupled with similar efforts to improve health service delivery so as to build trust in the formal health system. Our findings suggest the importance of fostering and leveraging existing social solidarity to increase health insurance enrolment. NHIF can leverage existing solidarity and willingness to prepay for healthcare to increase enrolment into health insurance. However, there is an urgent need to revise payment approaches away from flat rate contributions currently employed in Kenya for the informal sector to allow for adequate risk and income cross-subsidization. Finally, the government should consider revising or ensuring proper implementation of targeting policies to ensure subsidy programs benefit intended beneficiaries. In this way, we propose that a stronger NHIF will help Kenya more fully realize its UHC ambitions.

## Data availability statement

The raw data supporting the conclusions of this article will be made available by the authors, without undue reservation.

## Ethics statement

The studies involving human participants were reviewed and approved by Ethical approval was obtained from University of Witwatersrand (Certificate no. M210216) and the Moi University/Moi Teaching and Referral Hospital Institutional Research Ethics Committee (IREC)-(No. 0003935), before beginning the study. Additional approval was obtained from the National Commission for Science Technology and Innovation (NACOSTI-P/21/12262) and Busia County Health Department. The patients/participants provided their written informed consent to participate in this study.

## Author contributions

BM, JG, and AKi contributed to the conceptualization of the project and drafted the manuscript. BM, JG, Aki, and AKo contributed to the development of data collection materials. BM contributed to the recruitment of participants and data collection. All authors edited and approved the final draft. All authors contributed to the article and approved the submitted version.

## Funding

This work was funded by CARTA and AbbVie Foundation (Grant 341). BM was supported by the Consortium for Advanced Research Training in Africa (CARTA). CARTA is jointly led by the African Population and Health Research Center and the University of the Witwatersrand and funded by the Carnegie Corporation of New York (Grant No. G-19-57145), Sida (Grant No. 54100113), Uppsala Monitoring Center, Norwegian Agency for Development Cooperation (Norad), and by the Wellcome Trust [reference no. 107768/Z/15/Z] and the UK Foreign, Commonwealth and Development Office, with support from the Developing Excellence in Leadership, Training and Science in Africa (DELTAS Africa) programme. The statements made and views expressed are solely the responsibility of the Fellow. For the purpose of Open Access, the author has applied a CC BY public copyright license to any Author Accepted Manuscript version arising from this submission.

The Population Health Program in AMPATH under which this research was nested was supported by ABBVIE Foundation (Grant 341).

## Conflict of interest

The authors declare that the research was conducted in the absence of any commercial or financial relationships that could be construed as a potential conflict of interest.

## Publisher's note

All claims expressed in this article are solely those of the authors and do not necessarily represent those of their affiliated organizations, or those of the publisher, the editors and the reviewers. Any product that may be evaluated in this article, or claim that may be made by its manufacturer, is not guaranteed or endorsed by the publisher.

## References

[1] Mathauer I Torres LV Kutzin J Jakab M Hanson K. Pooling financial resources for universal health coverage: options for reform. Bull World Health Organ. (2020) 98:132–9. 10.2471/BLT.19.234153 32015584 PMC6986215

[2] Maarse H Paulus A. Has solidarity survived? A comparative analysis of the effect of social health insurance reform in four European countries. J Health Polit Policy Law. (2003) 28:585–614. 10.1215/03616878-28-4-585 12956517

[3] Cotlear D Nagpal S Smith O Tandon A Cortez R. Going Universal: How 24 Developing Countries Are Implementing Universal Health Coverage Reforms from the Bottom Up. (2015). 10.1596/978-1-4648-0610-0

[4] Kutzin J. Health financing for universal coverage and health system performance: concepts and implications for policy. Bull World Health Organ. (2013) 91:602–11. 10.2471/BLT.12.113985 23940408 PMC3738310

[5] World Health Organization. Health System Financing: The Path to Universal Coverage (2010). 10.2471/BLT.10.078741 PMC2878164 20539847

[6] The World Bank. Financing health in low-income countries. Heal Financ Revisit. (2005) 209–48. 10.1596/978-0-8213-0900-1

[7] World Bank. Informal Enterprises in Kenya [IInternet]. Washington, DC (2016). Available online at: https://openknowledge.worldbank.org/handle/10986/24973

[8] Douwes R Stuttaford M London L. Social solidarity, human rights, and collective action: considerations in the implementation of the National Health Insurance in South Africa. Heal Hum Rights J. (2018) 20:185–96. 30568412 PMC6293357

[9] Davies B Savulescu J. Solidarity and responsibility in health care. Public Health Ethics. (2019) 12:133–44. 10.1093/phe/phz008 31384302 PMC6655468

[10] Gualda E. Altruism, solidarity and responsibility from a committed sociology: contributions to society. Am Sociol. (2022) 53:29–43. 10.1007/s12108-021-09504-1 34376856 PMC8342653

[11] Mbogo BA Mcgill D. Perspectives on financing population- based health care towards Universal Health Coverage among employed individuals in Ghanzi district, Botswana: A qualitative study. BMC Health Serv Res. (2016) 1–14. 10.1186/s12913-016-1657-2 27543136 PMC4992196

[12] Snell K Tarkkala H Tupasela A. A solidarity paradox – welfare state data in global health data economy. Heal. (2021) 0. 10.1177/13634593211069320 34965751 PMC10423432

[13] Goudge J Akazili J Ataguba J Kuwawenaruwa A Borghi J Harris B. Social solidarity and willingness to tolerate risk- and income-related cross-subsidies within health insurance: experiences from Ghana, Tanzania and South Africa. Health Policy Plan. (2012) 27(Suppl.1):i55–63. 10.1093/heapol/czs008 22388501

[14] Dror DM Shahed Hossain SA Majumdar A Koehlmoos TLP John D Panda PK. What factors affect voluntary uptake of community-based health insurance schemes in low- and middle-income countries? A systematic review and meta-analysis. PLoS ONE. (2016) 11:160479. 10.1371/journal.pone.0160479 27579731 PMC5006971

[15] Zang L Wang H Wang L Hsiao W. Social capital and farmer's willingness-to-join a newly established community-based health insurance in rural China. Health Policy. (2006) 76:233–42. 10.1016/j.healthpol.2005.06.001 16046027

[16] Jost T. NHealth care access in the United States. Conflicting concepts of justice and little solidarityo title. Med Law. (2008) 27:605–16. 19004385

[17] Mathauer I Schmidt JO Wenyaa M. Extending social health insurance to the informal sector in Kenya. An assessment of factors affecting demand. Int J Health Plann Manage. (2008) 23:51–68. 10.1002/hpm.914 18050152

[18] Fusheini A. The politico-economic challenges of ghana's national health insurance scheme implementation. Int J Heal Policy Manag. (2016) 5:543–52. 10.15171/ijhpm.2016.47 27694681 PMC5010657

[19] Fenenga CJ Nketiah-Amponsah E Ogink A Arhinful DK Poortinga W Hutter I. Social capital and active membership in the Ghana National Health insurance scheme - a mixed method study. Int J Equity Health. (2015) 14:1–12. 10.1186/s12939-015-0239-y 26526063 PMC4630914

[20] Barasa E Rogo K Mwaura N Chuma J Barasa E Rogo K. Kenya national hospital insurance fund reforms: implications and lessons for universal health coverage kenya national hospital insurance fund reforms: implications and lessons for universal health coverage. Heal Syst Reform. (2018) 4:346–61. 10.1080/23288604.2018.1513267 30398396 PMC7116659

[21] Health Policy Plus. Is Kenya Allocating Enough Funds for Healthcare? (2021). Available online at: http://www.healthpolicyplus.com/ns/pubs/18441-18879_KenyaNCBABrief.pdf

[22] WHO. The Abuja Declaration: Ten Years On. Heal (San Fr (2011) (2000):1–4.

[23] Government of Kenya (GoK). Road to Universal Health Coverage. Kenya Yearbook Editorial Board Chief (2020).

[24] Chuma J Mulupi S McIntyre D. Providing Financial Protection and Funding Health Service Benefits for the Informal Sector: Evidence From Sub-Saharan Africa (2013).

[25] World Bank Group. Kenya Poverty and Equity Brief (2020).

[26] Government of Kenya (GoK). Kenya Vision 2030 (2006).

[27] Ministry of Health G of K. Kenya Health Financing Strategy Ministry of Health Overview of the Kenya Health Financing Strategy (2020).

[28] Okungu VR McIntyre D. Does the informal sector in Kenya have financial potential to sustainably prepay for health care? Implications for financing universal health coverage in low-income settings. Heal Syst Reform. (2019) 5:145–57. 10.1080/23288604.2019.1583492 30924731

[29] Barasa EW Mwaura N Rogo K Andrawes L. Extending voluntary health insurance to the informal sector: experiences and expectations of the informal sector in Kenya. Wellcome Open Res. (2017) 2:1–13. 10.12688/wellcomeopenres.12656.1 29387800 PMC5698913

[30] Ministry of Health G of K. Kenya Household Health Expenditure and Utilization Survey (2018).

[31] WHO. Strategic Purchasing for Universal Health Coverage: Key Policy Issues and Questions (2017).

[32] Creswell J. Qualitative, Quantitative, and Mixed Methods Approaches. SAGE Publications, Inc (2014).

[33] County Government of Busia. County Integrated Development Plan [Internet]. (2018). Available online at: https://www.devolutionhub.or.ke/resource/county-government-of-busia-health-sector-strategic-and-investment-plan-2018-2023

[34] Mercer T Gardner A Andama B Chesoli C Christoffersen-deb A Dick J. Leveraging the Power of Partnerships: Spreading the Vision for a Population Health Care Delivery Model in Western Kenya. (2018). p. 1–11. 10.1186/s12992-018-0366-5 29739421 PMC5941561

[35] World Health Organization. World Health Survey: Guide to Administration and Question by Question (2002).

[36] Ministry of Health G of K. (2013). Kenya Household Health Expenditure And Utilization Survey (2014).

[37] Gale K N Heath G Cameron E Rashid S Redwood S. Using the framework method for the analysis of qualitative data in multi-disciplinary health research. BMC, Med Res Methodol [Internet]. (2013) 7:260–1. Available online at: http://www.biomedcentral.com/1471-2288/13/117 24047204 10.1186/1471-2288-13-117 PMC3848812

[38] Koon AD. Framing Universal Health Coverage in Kenya: An Interpretive Analysis of Health Financing Politics. (2017). p. 25–44. Available online at: http://researchonline.lshtm.ac.uk/4398421/1/2017_PHP_PhD_Koon_AD.pdf 10.1093/heapol/czaa133 33227121

[39] Paugam S Cousin B Giorgetti C Naudet J. What do the Rich Think of the Poor? Seuil (2017). Available online at: https://www.sciencespo.fr/en/news/what-do-the-rich-think-of-the-poor (accessed Sep 1, 2022).

[40] Künzler D. The Politics of Health Care Reforms in Kenya and their Failure. Sozialpolitik Ch. (2016) 2016:1–20. 10.18753/2297-8224-64

[41] Munguti Dennis. Perceptions of Households Towards Health Insurance and Their Implication to Enrolment, Kenya (2020).

[42] Kabia E Mbau R Muraya KW Morgan R Molyneux S Barasa E. How do gender and disability influence the ability of the poor to benefit from pro-poor health financing policies in Kenya? An intersectional analysis. Int J Equity Health. (2018) 17:1–12. 10.1186/s12939-018-0853-6 30231887 PMC6146517

[43] Hanson K Brikci N Erlangga D Alebachew A De Allegri M Balabanova D. The Lancet Global Health Commission on financing primary health care: putting people at the centre. Lancet Glob Heal. (2022) 10:e715–72. 10.1016/S2214-109X(22)00005-5 35390342 PMC9005653

[44] Kabia E Mbau R Oyando R Oduor C Bigogo G Khagayi S. “We are called the et cetera”: Experiences of the poor with health financing reforms that target them in Kenya. Int J Equity Health. (2019). 18:1–14. 10.1186/s12939-019-1006-2 31234940 PMC6591805

[45] Chuma J Maina T Ataguba J. Does the distribution of health care benefits in Kenya meet the principles of universal coverage? BMC Public Health. (2012) 12:20. Available online at: http://www.biomedcentral.com/1471-2458/12/20 10.1186/1471-2458-12-20 22233470 PMC3280172

[46] Buigut S Ettarh R Amendah DD McGuire F Vijayasingham L Vassall A. Extending social health insurance to the informal sector in Kenya. An assessment of factors affecting demand. Int J Equity Health. (2019) 18:1–12. Available online at: http://www.biomedcentral.com/1472-6963/12/6630606218

[47] Falkingham J Namazie C. Measuring Health Poverty: A Review of Approaches to Identifying the Poor. (2002). p. 44. Available online at: http://eprints.soton.ac.uk/35016/

[48] Nsiah-boateng E Aikins M. Trends and characteristics of enrolment in the National Health Insurance Scheme in Ghana: a quantitative analysis of longitudinal data. Global Health Res Policy. (2018). 6:1–10. 10.1186/s41256-018-0087-6 30460332 PMC6233555

